# Supplementary material for: A Global Screen for Assembly State Changes of the Mitotic Proteome by SEC-SWATH-MS
Source: Cell Syst. 2020 Feb 26;10(2):133–155.e6. doi: 10.1016/j.cels.2020.01.001 (PMC7042714; doi:10.1016/j.cels.2020.01.001)

Q9UHQ9 | NB5R1_HUMAN | CYB5R1 NQO3A2 UNQ3049/PRO9865
Monomer MW [kDa]: 34.095 Monomer expected elution fraction: 50

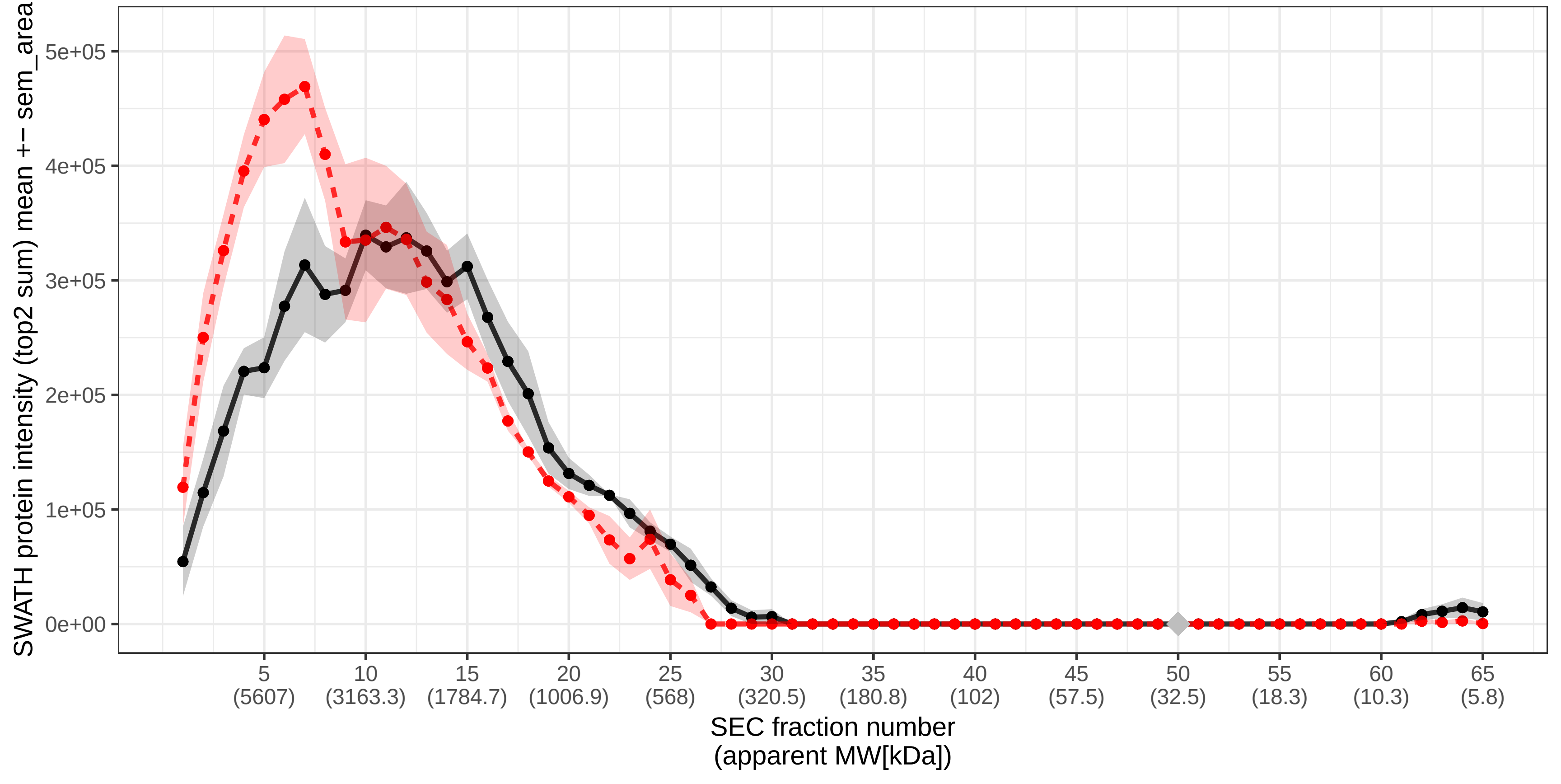

Supplement: Data S1. SEC-SWATH-MS Protein Chromatograms, Related to Figure 1 [file mmc6.zip › SECchrom_Q9UHQ9_NB5R1_HUMAN_CYB5R1_NQO3A2_UNQ3049_PRO9865.pdf]
